# Optometrische Schulreihenuntersuchungen: Erste epidemiologische Daten von Kindern und Jugendlichen der 5. bis 7. Klasse

**DOI:** 10.1007/s00347-021-01427-z

**Published:** 2021-06-10

**Authors:** Hakan Kaymak, Kai Neller, Birte Graff, Kristina Körgesaar, Achim Langenbucher, Berthold Seitz, Hartmut Schwahn

**Affiliations:** 1 Institut für Experimentelle Ophthalmologie, Universitätsklinikum des Saarlandes UKS, Homburg/Saar, Deutschland; 2 Internationale Innovative Ophthalmochirurgie, Breyer Kaymak Klabe Augenchirurgie, Düsseldorf, Deutschland; 3 Klinik für Augenheilkunde, Universitätsklinikum des Saarlandes UKS, Homburg/Saar, Deutschland; 4 Ernst-Abbe-Hochschule Jena, Jena, Deutschland

**Keywords:** Myopie, Biometrie, Achslänge, Schulkinder, Epidemiologie, Myopia, Biometry, Axial length, Schoolchildren, Epidemiology

## Abstract

**Hintergrund:**

Wiederkehrende Schulreihenuntersuchungen dienen dazu, Kinder und Jugendliche mit erhöhtem Achslängenwachstum der Augen zu identifizieren und dafür zu sensibilisieren, dass die richtige Sehhilfe und ausreichend Aufenthalt im Freien präventive Faktoren gegen die Entwicklung einer hohen Myopie sind. Die erhobenen biometrischen Daten helfen außerdem, die epidemiologischen Datenlage zur Schulmyopie zu erweitern, die schließlich eine Grundlage für die Auswahl von Therapieoptionen bilden.

**Material und Methoden:**

Mittels berührungsfreier Biometrie wurden Hornhautradius, zentrale Hornhautdicke, Vorderkammertiefe, Linsendicke und Achslänge der Augen ermittelt. Optische Kohärenztomographie (OCT) wurde zur Bestimmung der subfovealen Aderhautdicke eingesetzt. Zusätzlich wurden der mesopische und photopische Pupillendurchmesser bestimmt.

**Ergebnisse:**

Biometrische Daten der Augen konnten von 257 (Alter 11,2 ± 1,1 Jahre, 31,9 % [82] weiblich, 68,1 % [175] männlich) der untersuchten 274 Schulkinder erhoben werden. Es zeigten sich ein mittlerer Hornhautradius (Mittelwert [MW] ± Standardabweichung [SD], weiblich/männlich) von 7,74 ± 0,23/7,89 ± 0,22 mm, zentrale Hornhautdicke von 556,80 ± 31,31/565,68 ± 33,12 µm, Vorderkammertiefe von 3,62 ± 0,28/3,71 ± 0,25 mm, Linsendicke von 3,48 ± 0,18/3,46 ± 0,17 mm sowie Achslänge von 23,03 ± 0,88/23,51 ± 0,88 mm. Die subfoveale Aderhautdicke konnte bei 240 Schulkindern ausgewertet werden und betrug 335,12 ± 60,5 µm. Die gemessene mesopische und photopische Pupillengröße betrug 6,38 ± 0,70 mm bzw. 3,11 ± 0,63 mm.

**Diskussion:**

Die ermittelten Achslängen der Augen stimmen mit den Normwerten bei europäischen Kindern überein. Es zeigt sich ein Unterschied in der Biometrie zwischen weiblichen und männlichen Augen. Die geplanten Wiederholungsuntersuchungen werden das Erstellen von ersten Wachstumskurven ermöglichen.

## Abstract (en)

**Background:**

Annually recurring optometric screening helps to identify children with increased axial growth and also to create awareness for wearing properly corrected glasses and for spending enough time outdoors, both of which are crucial for healthy eyes. The obtained biometric data help to expand the epidemiological information on myopia in schoolchildren, which is fundamental for the selection of the correct treatment.

**Material and methods:**

Contact-free biometry of the eye was used to assess central corneal thickness, anterior chamber depth, lens thickness and axial length. Central choroidal thickness was manually assessed using optical coherence tomography (OCT). In addition, the mesopic and photopic pupil sizes were measured.

**Results:**

Biometric data were obtained from 257 (mean age 11.2 ± 1.1 years, 31.9% female, *n* = 82, 68.1% male, *n* = 175) out of a total of 274 examined children. Mean corneal radius (mean ± SD, female/male) was 7.74 ± 0.23 mm/7.89 ± 0.22 mm, central corneal thickness was 556.80 ± 31.31 µm/565.68 ± 33.12 µm, anterior chamber depth was 3.62 ± 0.28 mm/3.71 ± 0.25 mm, lens thickness was 3.48 ± 0.18 mm/3.46 ± 0.17 mm and axial length was 23.03 ± 0.88 mm/23.51 ± 0.88 mm. Choroidal thickness was assessed in 240 children and was 335.12 ± 60.5 µm. Mesopic and photopic pupil sizes were 6.38 ± 0.70 mm and 3.11 ± 0.63 mm, respectively.

**Conclusion:**

The axial lengths found are consistent with the normal values for European children. A difference between male and female eyes could be observed. The repetition of these examinations in the future will enable the generation of growth charts.

In der Literatur ist das Einsetzen der Schulmyopie ab dem 6. Lebensjahr beschrieben. Für Kinder und Jugendliche macht sich eine Myopie durch einen unscharfen Seheindruck in der Ferne bemerkbar. Die Dokumentation der Biometrie von Schulkindern über mehrere Jahre liefert wertvolle Erkenntnisse darüber, welche Teile des Auges an dem Prozess der Myopieprogression beteiligt sind. Eltern und Lehrern kann so verdeutlicht werden, welche pathologischen Veränderungen hinter dem von den Kindern wahrgenommenen unscharfen Sehen in der Ferne stecken. Die erhobenen Daten tragen zudem zur epidemiologischen Datenerhebung bei und helfen bei dem Erstellen von Nomogrammen zur besseren Einschätzung des Myopierisikos für Kinder und Jugendliche aus dem europäischen Raum.

## Einleitung

Nachdem wir in unserer vorangegangenen Publikation [8] über die logistische Machbarkeit von Schulreihenuntersuchungen berichteten, möchten wir in dieser Arbeit weiter über die erhobenen biometrischen Daten berichten. Der thematische Schwerpunkt unserer ersten Arbeit lag auf der Refraktionsbestimmung in Reihenuntersuchungen und dem daraus entstehenden Mehrwert für die Schulkinder. Es wurde über die unterschiedlichen Ergebnisse zwischen objektiver und subjektiver Refraktion sowie über den Einsatz von Anamnesebögen zum Abschätzen der mit einer Myopie in Zusammenhang stehenden Risikofaktoren berichtet.

Wie gezeigt, spielt bei der Schulmyopie der geringe Aufenthalt im Freien eine große Rolle [8]. Davon abzugrenzen ist eine Myopie, bei welcher Umweltfaktoren keinen direkten Einfluss auf die vorliegende Myopieprogression im Kindes- und Jugendalter nehmen, da hier die Entwicklung einer Myopie primär durch genetische Faktoren begründet ist [24].

Das physiologische Augenwachstum für im Erwachsenenalter emmetrope Kinder und Jugendliche wird in Bezug auf das Achslängenwachstum mit 0,1 mm/Jahr für 6‑ bis 14-Jährige angegeben [12], mit welchem auch eine vertikale und horizontale Vergrößerung des Auges und eine Zunahme des Hornhautdurchmessers verbunden ist, welches zu einer Abnahme der Hornhautbrechkraft führt [5]. Bei diesem Prozess verändert sich der Refraktionsstatus der Augen von einer leichten Hyperopie zu einer Emmetropie, welche in der Literatur für einen Bereich des sphärischen Äquivalents (SÄ) von −0,50 < SÄ ≤ +0,5 dpt beschrieben wird. Zur Emmetropisierung trägt auch eine Veränderung der Augenlinse bei, welche sich im Wachstumsprozess des Auges abflacht [13].

Bei der Entwicklung einer Myopie ist das Augenwachstum erhöht; die gemessene Achslänge ändert sich durchschnittlich um mehr als 0,1 mm/Jahr, wobei hier bei der Interpretation einer Myopieprogression zu beachten gilt, dass Tideman et al. [21] eine Zunahme von 0,19 mm/Jahr bei 9‑Jährigen nicht als pathologisch sehen.

Wird die Myopieprogression andererseits über den Refraktionsstatus beschrieben, so haben Rozema et al. [17] gezeigt, dass die Abnahme der Linsendicke bei gerade myop werdenden Kindern stärker ist als bei emmetropen Kindern. Mit der Abnahme der Linsendicke ist eine Abnahme der Linsenbrechkraft verbunden, wodurch das Einsetzen der Myopie herausgezögert wird [7].

Wie auch von Brennan et al. [1] beschrieben, kann die Dokumentation der Zunahme der Achslänge aussagekräftiger sein als das alleinige Erheben der Refraktion. Allerdings ist hierzu das Erstellen von Nomogrammen wie bei Tideman et al. [21] notwendig, um altersspezifisch ein zu hohes Augenwachstum unabhängig von dem aktuellen Refraktionsstatus des Auges erkennen zu können. Unsere hier vorgestellte Arbeit verfolgt das Ziel, weitere Normdaten für den westeuropäischen Raum zur Verfügung zu stellen.

Um der Entwicklung einer hohen Myopie vorzubeugen, gibt es inzwischen spezielle Brillengläser [9, 18], multifokale Kontaktlinsen [23] sowie die pharmakologische Therapie mit Atropin [3]. Es ist aus asiatischen Studien zur Hemmung der Myopieprogression bei Kindern und Jugendlichen bekannt, dass Atropin-Augentropfen eine direkte Auswirkung auf den Pupillendurchmesser und die Aderhautdicke des Auges haben [10, 25]. Hier können die im Rahmen des Pilotprojektes erhobenen Werte dazu beitragen, Normdaten für das Therapiemanagement für europäische Kinder bereitzustellen.

## Material und Methoden

Alle Untersuchungen wurden mit Zustimmung der Ethikkommission (Nr.: 2019/1520), im Einklang mit nationalem Recht sowie gemäß der Deklaration von Helsinki von 1975 durchgeführt. Von allen beteiligten Schulkindern und deren Eltern liegt eine Einverständniserklärung vor.

### Durchführung der ersten Datenerhebung

**Figure 1:**
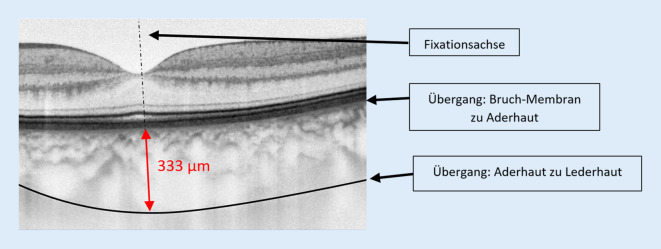

Von September bis November 2019 fanden die ersten Vollerhebungen der 5. bis 7. Jahrgangstufen an einem staatlichen Gymnasium im Raum Düsseldorf statt. Neben der zuvor beschriebenen Erhebung der objektiven und subjektiven Refraktionswerte [8] wurden mittels Biometer (IOL-Master 700, Zeiss, Oberkochen, Deutschland) an beiden Augen die Achslänge, Linsendicke, Vorderkammertiefe, zentrale Hornhautdicke sowie die vorderen Hornhautradien gemessen. Zur Bestimmung der subfovealen Aderhautdicke erfolgte ein hochauflösender B‑Scan der Makula (Cirrus 5000, Zeiss, Oberkochen, Deutschland). Die Aderhautdicke wurde manuell mit der Cirrus Software (Zeiss, Oberkochen, Deutschland) ausgewertet (Vorgehen s. Abb. 1). Die subfoveale Aderhautdicke wurde auch durch einen zweiten Autor bestimmt. Bei der Interraterreliabilität betrug der Intraklassenkorrelationskoeffizient 0,89.

Der mesopische (4 Lx) und der photopische (300 Lx) Pupillendurchmesser wurden mittels Videopupillographie (Aladdin, Topcon, Tokyo, Japan) ermittelt.

### Statistische Analyse

Der Pearson-Korrelationskoeffizient für lineare Zusammenhänge und die Regressionsgerade wurden mit Matlab Version 2020b (MathWorks, Natick, MA, USA) berechnet. Der t‑Test für unabhängige Stichproben zum Testen auf Unterschiede zwischen männlichen und weiblichen Augen wurde mit der Software Excel (Microsoft, Redmond, WA, USA) durchgeführt. Für die statistische Auswertung wurden die Daten der rechten Augen verwendet.

## Ergebnisse

Im Herbst 2019 nahmen 274 Schulkinder freiwillig an den Untersuchungen teil. Die biometrischen Daten von 257 Schulkindern (11,2 ± 1,1 Jahre) der 5. bis 7. Klasse konnten erhoben werden. Bei 17 Schulkindern (6,2 %) konnten die biometrischen Daten aufgrund von schlechter Fixation während der Messung und der damit verbundenen unvollständigen und fehlerbehafteten Erfassung der Messwerte in der Software des Geräts nicht ausgewertet werden. Die subfoveale Aderhautdicke mittels OCT konnte bei 240 Schulkindern ausgewertet werden. Bei 34 Schulkindern (12,4 %) konnte aufgrund unruhiger Fixation kein klarer OCT-B-Scan erfolgen. Bei 270 Schulkindern konnte die Pupillengröße unter photopischen und mesopischen Lichtbedingungen gemessen werden. Hier waren es lediglich 4 (1,46 %) Schulkinder, welche aufgrund von schlechter Fixation in der Pupillometrie nicht vermessen werden konnten.

In den Tab. 1 und 2 sind die gemessenen Parameter für die Schülerinnen und Schüler in Abhängigkeit des Alters dargestellt.

**Table 1:** 

Alter (Jahre)	Anzahl	Mittlerer Hornhautradius in mm	Zentrale Hornhautdicke in µm	Vorderkammertiefe in mm	Linsendicke in mm	Achslänge in mm
9	2	7,78/8,31	545/541	3,31/3,36	3,62/3,42	22,64/23,98
10	24	7,71 ± 0,19	546,50 ± 24,99	3,66 ± 0,24	3,45 ± 0,16	22,95 ± 0,49
11	22	7,70 ± 0,22	557,86 ± 27,62	3,61 ± 0,36	3,47 ± 0,25	22,76 ± 1,05
12	29	7,77 ± 0,24	561,55 ± 36,34	3,62 ± 0,25	3,50 ± 0,16	23,27 ± 0,86
13 bis 14	5	7,76 ± 0,25	579,60 ± 36,12	3,52 ± 0,11	3,51 ± 0,16	23,18 ± 1,46

**Table 2:** 

Alter (Jahre)	Anzahl	Mittlerer Hornhautradius in mm	Zentrale Hornhautdicke in µm	Vorderkammertiefe in mm	Linsendicke in mm	Achslänge in mm
9	1	7,77	562,00	3,98	3,27	23,60
10	56	7,82 ± 0,21	563,41 ± 38,13	3,69 ± 0,26	3,47 ± 0,19	23,23 ± 0,80
11	50	7,92 ± 0,21	564,68 ± 30,91	3,74 ± 0,25	3,42 ± 0,16	23,57 ± 0,78
12	55	7,93 ± 0,22	565,71 ± 30,41	3,69 ± 0,25	3,48 ± 0,18	23,65 ± 1,03
13	9	7,84 ± 0,24	575,22 ± 28,61	3,73 ± 0,30	3,49 ± 0,16	23,83 ± 0,69
14 bis 16	4	7,92 ± 0,17	589,00 ± 37,55	3,76 ± 0,34	3,48 ± 0,12	24,04 ± 0,93

In Tab. 3 sind die biometrischen Daten der weiblichen und männlichen Augen gegenübergestellt. Das Testen auf Normalverteilung der einzelnen Parameter mittels Kolmogorov-Smirnov-Test ergab, dass alle Parameter beider Geschlechter normalverteilt sind. Mittels t‑Test wurde auf Unterschiede zwischen den einzelnen Parametern getestet (Signifikanzniveau: *p < 0,05, **p < 0,01, ***p < 0,001).

**Table 3:** 

Geschlecht	Anzahl	Mittlerer Hornhautradius in mm	Zentrale Hornhautdicke in µm	Vorderkammertiefe in mm	Linsendicke in mm	Achslänge in mm
Weiblich	82	7,74 ± 0,23	556,80 ± 31,31	3,62 ± 0,28	3,48 ± 0,18	23,03 ± 0,88
Männlich	175	7,89 ± 0,22***	565,68 ± 33,12*	3,71 ± 0,25**	3,46 ± 0,17	23,51 ± 0,88***

**Figure 2:**
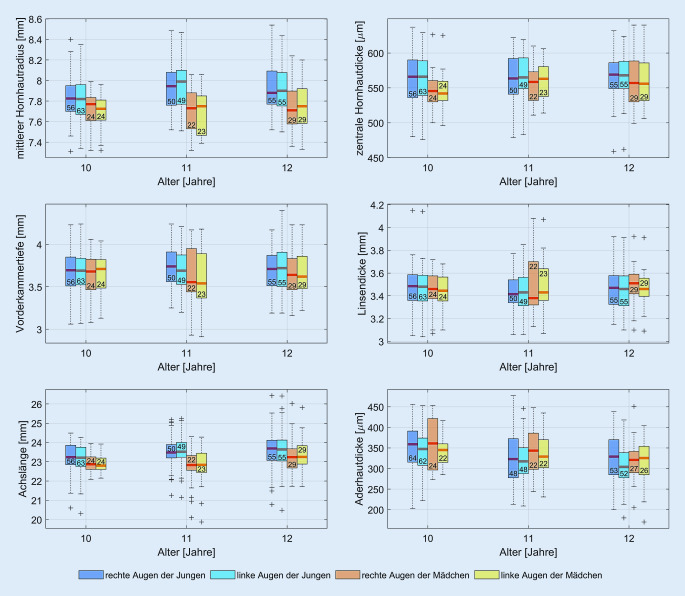

In Abb. 2 sind die mit dem Biometer gemessenen Parameter dargestellt. Aufgrund des geringen Stichprobenumfangs in den Altersklassen 9 sowie 13 bis 16 sind in Abb. 2 die Altersklassen von 10 bis 12 Jahren ausgewertet.

**Figure 3:**
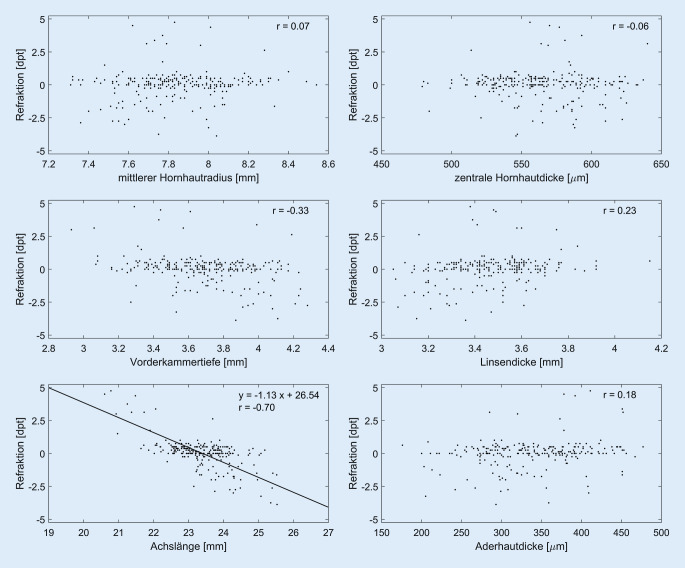

Die Abb. 3 zeigt die gemessene Refraktion in Abhängigkeit von den erhobenen Parametern mittlerer Hornhautradius, Hornhautdicke, Vorderkammertiefe, Linsendicke und Achslänge. Bei 184 der 257 untersuchten Schulkinder wurde neben der objektiven Refraktion auch die subjektive Refraktion ermittelt, sodass diese, wenn vorhanden, zur Auswertung der in Abb. 3 gezeigten Daten verwendet wurde. In den restlichen 73 Fällen wurden die Werte der objektiven Refraktion verwendet.

Wird der gesamte Refraktionsbereich betrachtet, so kann die Korrelation zwischen Achslänge und Refraktion (r = −0,70) durch die Regressionsgerade SÄ[dpt] = −1,13 AL + 26,54 beschrieben werden (Achslänge = AL). Über einen Refraktionsbereich von −3 bis 3 dpt sinkt der Korrelationskoeffizient auf −0,48, und die Regressionsgerade ändert sich zu SÄ[dpt] = −0,54 AL + 12,60.

### Subfoveale Aderhautdicke

Es zeigt sich keine Korrelation zwischen der subfovealen Aderhautdicke und der Refraktion (Abb. 3). Die subfoveale Aderhautdicke beträgt (MW ± SD) insgesamt 335,12 ± 60,5 μm und aufgeteilt nach Geschlecht (weiblich/männlich) 342,92 ± 59,19/331,73 ± 60,87 µm.

### Pupillendurchmesser

**Figure 4:**
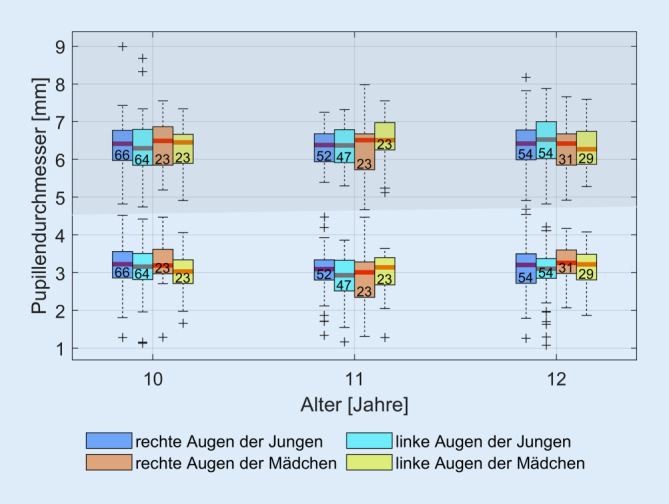

Der mesopische Pupillendurchmesser beträgt insgesamt 6,38 ± 0,70 mm und aufgeteilt nach Geschlecht (weiblich/männlich) 6,31 ± 0,68/6,41 ± 0,72 mm. Der photopische Pupillendurchmesser beträgt 3,11 ± 0,63 mm und aufgeteilt nach Geschlecht (weiblich/männlich) 3,12 ± 0,68/3,10 ± 0,61 mm. In Abb. 4 sind der gemessene mesopische und photopische Pupillendurchmesser nach Altersklassen (10 bis 12 Jahre) in Boxplotdiagrammen dargestellt.

## Diskussion

Im Rahmen dieser ersten Messserie unserer Pilotstudie zu optometrischen Schulreihenuntersuchungen konnten die 5. bis 7. Jahrgangsstufe eines Gymnasiums erfolgreich vermessen werden. Aufgrund der COVID-19-Pandemie und den damit verbundenen anhaltenden Restriktionen an den Schulen können die Folgemessungen und die damit verbundene Auswertung zur Änderung der erhobenen Biometrie und Refraktion an den Schulen derzeit noch nicht stattfinden. Es ist geplant, das Projekt Ende 2021 fortzuführen. Dann werden neben neuen Erstuntersuchungen in den unteren Klassen auch die Folgemessungen an den im Herbst 2019 untersuchten Kindern stattfinden können.

### Umfangreiche Erhebung der Biometrie

In aktuellen europäischen Studien zur Schulmyopie wurden neben Refraktion und Brillenwerten auch biometrische Daten der Augen wie der Hornhautradius und die Achslänge veröffentlicht, da diese neben der Refraktion für das Beschreiben einer Myopie relevante Parameter sind [11, 19, 21]. Studien aus Asien und Amerika erhoben zusätzlich auch die Linsendicke und die damit verbundene Änderung der Linsenbrechkraft [17, 26]. Eine Einordnung unserer Daten in Bezug auf Vorderkammertiefe, Linsendicke und Hornhautradien in die Literatur ist schwierig, da keine vergleichbare Altersgruppe in der Literatur gefunden werden konnte. Asiatische Studien können generell schwer zum Vergleich herangezogen werden, da asiatische Augen sich in der Biometrie des vorderen Augenabschnitts von europäischen Augen unterscheiden [15].

**Figure 5:**
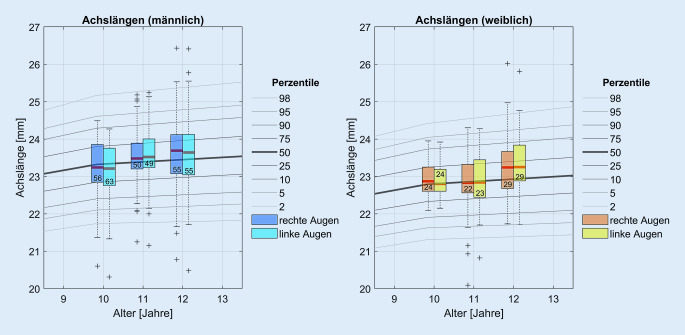

Die gemessenen Achslängen der Augen bestätigen die Normdaten von Tideman et al. [21] zu europäischen Kindern gleicher Altersgruppen, die anhand der Daten aus der Generation R, ALSPAC und RS-III-Studien Nomogramme zur Darstellung der Häufigkeitsverteilung der Augenlängen in der Population in Abhängigkeit des Alters erstellten [21]. So liegen die Mediane für die aktuell gemessenen Achslängen der 10- bis 12-jährigen Mädchen und Jungen in etwa auf der 50 %-Perzentile des Nomogramms aus Tideman et al. [21] (s. Abb. 5).

Die in Abb. 3 gezeigten erhobenen biometrischen Daten zeigen primär bei der gemessenen Achslänge einen Zusammenhang zwischen der Refraktion und der Achslänge (Pearson-Korrelationskoeffizient von 0,7). Dies zeigt, dass die durch eine Myopie verbundenen refraktiven Veränderungen primär durch ein Wachstum des hinteren Augenpols verursacht werden.

Es findet sich der aus der Literatur bekannte Unterschied, dass in derselben Altersklasse Augen von Jungen signifikant länger sind als die von Mädchen [21, 22].

Alle Messungen wurden ohne die Verabreichung von Zykloplegika durchgeführt. Es kann nicht ausgeschlossen werden, dass manche Kinder bei der Messung im teilweisen akkommodierten Zustand gemessen wurden. Dies kann im Hinblick auf die Auswertung der Biometrie zu einer leicht kleineren gemessenen Vorderkammertiefe und zu einer leicht höheren gemessenen Linsendicke geführt haben, da sich der vordere und hintere Radius der Augenlinse bei Akkommodation verkleinert, was die Mittendicke der Linse erhöht. Bezogen auf die in [8] gezeigten Refraktionswerte der objektiven und subjektiven Refraktion, hat sich gezeigt, dass ein Autorefraktometer aufgrund der Akkommodationsfähigkeit der Kinder die gemessenen Refraktionswerte mit einem mehr negativen Wert im Vergleich zur subjektiven Refraktion angeben kann. Hinzu kommt, dass die Wiederholgenauigkeit der Messung mit einem Autorefraktometer mit ±0,50 dpt angegeben wird [4].

Das Einsetzen der Myopie bei Kindern kann nur durch die Refraktion beurteilt werden, das Fortschreiten der Myopie aber am besten durch das regelmäßige Messen der Achslänge. Der Grund hierfür ist folgender: Hinsichtlich der Beurteilung und Interpretation der gemessenen Myopieprogression verweisen Brennan et al. [1] darauf, dass der Zusammenhang zwischen der Refraktion und der Achslänge nicht linear ist. So ist eine Änderung von 2,7 dpt/mm nur für eine Achslängenänderung bei einem Ausgangswert von 23 mm zulässig. Bei gleichbleibendem vorderem Augenabschnitt ändert sich die Refraktion bei einer Zunahme um 1 mm bei einem Auge von 30 mm Länge um weniger als 1,3 dpt/mm. So ist das Messen der Achslänge hinsichtlich der Interpretation der Myopieprogression weniger fehlerbehaftet als das Erheben der Refraktion. Die Messung der Achslänge mit dem IOL-Master 700 wird mit einer Wiederholgenauigkeit von ±0,01 mm (95 %-Konfidenzintervall) angegeben, was einer Genauigkeit in der Refraktion von ±0,03 dpt entspricht [2].

Wir plädieren für eine Ergänzung der Vorsorgeuntersuchungen für Kinder und Jugendliche um eine spezielle Myopievorsorgeuntersuchung. Ein erster Schritt könnte das Messen der Biometrie der Augen in der U9 und J1-Untersuchung zur Früherkennung von Krankheiten bei Kindern und Jugendlichen sein, allerdings sind diese Untersuchungen schon recht umfangreich, und es bedürfte einer zusätzlichen Schulung der Allgemeinärzte.

### Auswertung der Aderhautdicke

Anhand der Daten der Folgemessungen gilt es in Zukunft zu überprüfen, ob mit einem Achslängenwachstum auch eine Abnahme in der subfovealen Aderhautdicke einhergeht [16]. Unsere Daten zeigen, dass die subfoveale Aderhautdicke bei Kindern und Jugendlichen dicker ist (335,12 ± 60,5 μm bei 11,2 ± 1,1 Jahren) als bei Erwachsenen (271,7 ± 67,6 μm bei 45 ± 16 Jahren [14]).

Das Durchführen eines B‑Scans der Makularegion der Netzhaut bei der ersten Untersuchung der Kinder reicht aus, um mögliche Pathologien auszuschließen. Die Befundung der Aufnahme durch einen Arzt im Anschluss an die Untersuchung ist jedoch sehr zeitintensiv. Hier ist in Zukunft eine durch künstliche Intelligenz gestützte Vorbefundung wie bei Shah et al. [20] denkbar. Für die automatische Auswertung biometrischer Daten aus der OCT-Messung, besonders der Aderhautdicke, ist zurzeit noch keine in die Geräte integrierte Software verfügbar, sodass auf eine manuelle Auswertung zurückgegriffen werden muss, die sehr zeitintensiv ist und sich für Schulreihenuntersuchungen nicht eignet.

Es erscheint sinnvoll, in Zukunft auch den parapapillären Bereich der Netzhaut bei den Untersuchungen mit zu dokumentieren, da insbesondere hier Veränderungen der Netzhaut in Zusammenhang mit der Myopieprogression zu beobachten sind [6]. Am besten geeignet ist ein Fundusfoto der Netzhaut, wobei ein Gerät, wie beispielsweise das Triton (Fa. Topcon), besonders geeignet zu sein scheint, da dieses die Fundusfotografie und OCT-Aufnahme vereint.

### Auswertung der Pupillengröße

Der gefundene mittlere mesopische und photopische Pupillendurchmesser liegt bei 6,38 ± 0,70 mm und 3,11 ± 0,63 mm. Zum Vergleich liegen der mesopische und photopische Pupillendurchmesser der asiatischen Vergleichsgruppe von Yam et al. [25], welche unter gleichen Lichtbedingungen gemessen wurden, bei 6,66 ± 0,69 mm und 3,75 ± 0,82 mm. Die Werte der europäischen Kinder und der asiatischen Kinder stimmen also praktisch überein.

## Fazit für die Praxis

Die durch ein Biometer erhobene Achslänge zeigt einen direkten Zusammenhang zwischen der Länge der Augen und der Refraktion und ist ein wertvolles Instrument, welches unabhängig von der Akkommodation Informationen zum Wachstumsstatus des Auges liefern kann.
